# The Root Development Genes (RDGs) Network in *Brassica napus* and the Role of *BnaSHR-6* in Response to Low Nitrogen

**DOI:** 10.3390/plants14121842

**Published:** 2025-06-15

**Authors:** Xingying Chen, Sining Zhou, Shuang Ye, Zhuo Chen, Zexuan Wu, Shiying Liu, Liping Hu, Xiwen Yang, Xiaoya Yang, Peiji He, Xingzhi Qian, Huafang Wan, Ti Zhang, Nengwen Ying, Huiyan Zhao, Jiana Li, Cunmin Qu, Hai Du

**Affiliations:** 1 College of Agronomy and Biotechnology, Southwest University, Chongqing 400715, China; 13372700553@163.com (X.C.); zhousining1206@163.com (S.Z.); 17823360392@163.com (S.Y.); chenzhuo0405@163.com (Z.C.); w1045123799@126.com (Z.W.); liushiying08@163.com (S.L.); lipinghu0630@163.com (L.H.); yxw20000910@163.com (X.Y.); 13118406296@163.com (X.Y.); helium71@163.com (P.H.); qxz799252@163.com (X.Q.); wanhua05@163.com (H.W.); zhangti@swu.edu.cn (T.Z.); nwyin80@swu.edu.cn (N.Y.); zhaohuiyan@swu.edu.cn (H.Z.); ljn1950@swu.edu.cn (J.L.); 2 Academy of Agricultural Sciences, Southwest University, Chongqing 400715, China

**Keywords:** *Brassica napus* L., root, gene network, evolution, *BnaSHR-6*

## Abstract

The root system is vital for *Brassica napus* water/nutrient uptake and anchorage, highlighting the importance of identifying root development genes (RDGs). In this study, we identified 218 RDGs in *B. napus* through homology-based retrieval. Phylogenetic analysis of 22 representative species revealed that the RDGs are widely present in plants ranging from aquatic algae to angiosperms. RDGs in *B. napus* expanded through whole-genome duplication (WGD) events between *Brassica rapa* and *Brassica oleracea* ancestors and smaller duplications specific to *B. napus*. Promoter analysis identified 115 *cis*-elements, mainly abiotic stress-related and light-responsive. Transcription factor networks showed regulation by BBR-BPC, MIKC_MADS, AP2, and GRAS families. Transcriptome analysis under multiple stresses revealed that low nitrogen (LN) induced the most pronounced changes, with >50% (109/218) of RDGs differentially expressed in roots. Furthermore, we screened the *BnaSHR-6* gene, which is co-localized in both primary roots (PR) and lateral roots (LR), and responds strongly to LN. Phenotypic analysis revealed that the *BnaSHR-6* gene regulates the growth and development of both PR and LR under LN conditions, and confers a degree of resistance. These findings advance our understanding of RDGs in *B. napus* and provide valuable gene resources for subsequent molecular breeding.

## 1. Introduction

Root systems serve as the primary entry point for plants to acquire essential nutrients and provide mechanical stability [1,2]. Under nutrient deficiency or abiotic stresses such as soil salinization, roots exhibit adaptive responses [3,4]. During the development of plant roots, a multitude of genes play crucial roles in regulating processes such as root initiation, elongation, branching, cell differentiation, and responses to environmental stimuli. These genes are collectively referred to as Root Development Genes (RDGs) [5]. Therefore, the systematic identification of RDGs is crucial for improving crop stress tolerance and enhancing resource utilization efficiency.

There are two main types of root systems in plants: the taproot systems of dicots and the fibrous root systems of monocots [6]. The fibrous root system consists of a dense network of adventitious roots originating from the stem [7]. However, research on this architecture is limited, primarily focusing on Poaceae species such as rice and maize [8,9,10,11]. In taproot systems, the primary root (PR) serves as the main root, with branching consisting of smaller lateral roots (LR) and root hairs (RH) [12]. Current root research predominantly centers on taproot systems, particularly in the model plant *Arabidopsis*. The development of the primary root in *Arabidopsis* is driven by the root apical meristem (RAM), where stem cells maintained by the WOX5-dependent quiescent center drive growth [13,14,15,16]. LR development involves a regulatory network centered on *ARF7/19* alongside transcription factors like PLTs, SCR, and SHR [17,18,19]. RH formation in *Arabidopsis* is determined by cell-position specificity and controlled by R2R3-MYB/bHLH/WD40 complexes [20,21,22,23]. These studies in *Arabidopsis* have provided a framework for understanding conserved molecular mechanisms in plant root development and laid the foundation for studying RDGs in related crops.

As a representative taproot plant of agricultural importance, *Brassica napus* (genome AACC, 2n = 38) is a globally cultivated oilseed crop with significant value for vegetable consumption, edible oil production, and animal feed [24,25,26]. However, current research on *B. napus* has predominantly focused on aboveground traits, while root system research lags significantly, with existing work primarily addressing nutrient uptake and stress resistance [27,28,29], leaving RDG networks and developmental mechanisms poorly explored. Therefore, constructing a network of RDGs in *B. napus* is of great significance to elucidate the mechanisms underlying root development and to provide guidance for high-yield breeding.

In this study, we systematically analyzed the network of RDGs in *B*. *napus* at the genome-wide level, providing insights into the distribution, evolution, regulation, and expression patterns of RDGs. Additionally, we analyzed the expression patterns of RDGs under soil environmental stresses. *BnaSHR-6* stands out for its dual role in PR and LR development and its sustained up-regulation. This gene exhibits positive regulation under normal conditions and demonstrates an active response to low nitrogen (LN) stress. We subsequently cloned its coding sequence, generating transgenic lines to confirm its positive regulation of root architecture under LN. This study provides a thorough overview of the distribution, evolution, and expression patterns of RDGs in *B. napus*, alongside the functional validation of candidate genes, thereby establishing a robust foundation for future functional analyses of RDGs.

## 2. Results

### 2.1. Identification of RDGs in B. napus

**Figure 1:**
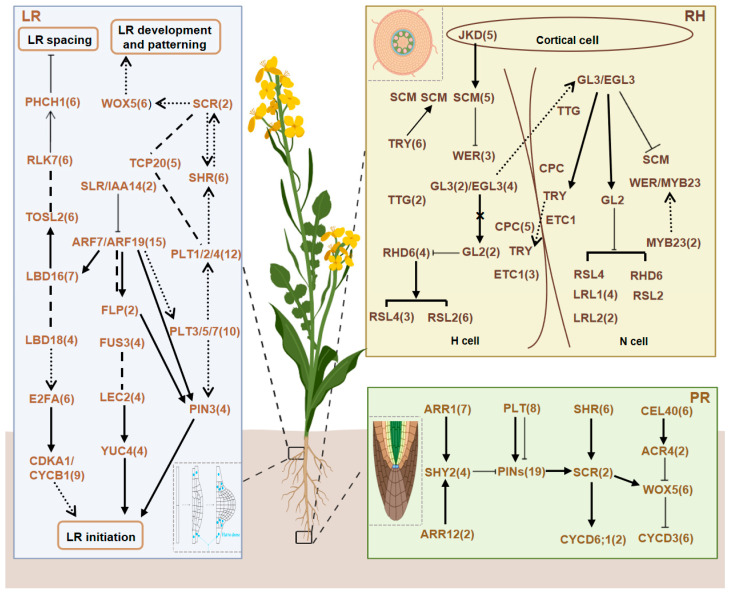
Primary roots (PR), lateral roots (LR), and root hairs (RH) gene networks of RDGs in *B. napus*. The numbers following the gene name indicate the gene number of the homologous gene in *B. napus*.

Based on the RDGs in *Arabidopsis* and the genomic data of *B. napus* publicly available on the BnIR online website (http://cbi.hzau.edu.cn/cgi-bin/bnapus/gb2/gbrowse/ZS11v0/, accessed on 1 September 2022), we identified a total of 218 RDGs in *B. napus*. Among these, 70 genes were associated with the PR network, 116 with the LR network, and 58 with the RH network (Figure 1 and Table S1). Notably, 26 genes overlapped between the PR and LR networks, including 14 genes involved in apical meristem growth (e.g., *BnaWOX5s* and *BnaSHRs*) and 12 genes regulating auxin concentration (e.g., *BnaPIN3s* and *BnaPLT1s*). The 70 candidate genes in the PR network originated from 17 gene families, including 7 TF families. The LR network comprised 116 candidate genes from 26 families (19 TF families), while the RH network included 58 candidate genes from 16 families (14 TF families).

Subcellular localization analyses indicated that proteins associated with root development in *B. napus* are predominantly nuclear-localized, suggesting that nuclear regulation plays a central role in the development of the root system in this species. Additionally, regulatory proteins were mainly localized in the nucleus, whereas functional proteins were distributed in non-nuclear subcellular structures. Members of the same protein family consistently exhibited identical subcellular localization patterns (Figure S1). Chromosomal localization results demonstrated that RDGs were evenly distributed between the A_n_ (106 genes) and C_n_ (108 genes) subgenomes. However, there was an uneven distribution across individual chromosomes and a higher concentration of genes at the chromosome ends (Figure S2).

### 2.2. Evolutionary Process of RDGs in the Plant Kingdom

**Figure 2:**
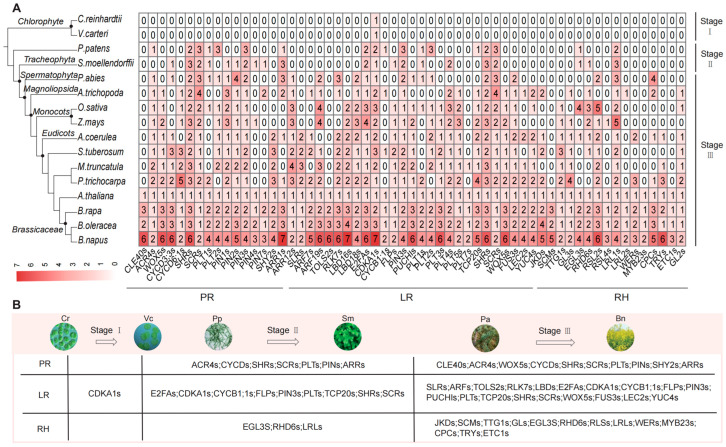
The distribution of RDGs in 16 typical plant species. (**A**) Homologous genes are marked at the bottom. The number in each square represents the number of genes. (**B**) The evolution of RDGs in 16 typical plant species. The evolution trend is divisible into three stages (Stage I, Stage II, and Stage III). Stage I is *Chlamydomonas reinhardtii* (Cr) to *Volvox carteri* (Vc), which contains only homologous genes of CDKA1. Stage II is *Physcomitrella patens* (Pp) to *Selaginella moellendorffii* (Sm), with the transition from aquatic to terrestrial plants and the initial formation of the RGD network. Stage III is *Picea abies* (Pa) to *Brassica napus* (Bn), where the number of RGDs further increased.

To investigate the distribution pattern of RDGs across the plant kingdom, we selected 16 representative species spanning major plant lineages, from aquatic green algae to angiosperms. From these species, 979 RDGs were identified, with an overall trend of amplification from algal plants to angiosperms. Based on phylogenetic distribution patterns, the evolutionary trajectory of RDGs can be categorized into three distinct stages (Figure 2 and Table S2). In the first stage, aquatic green algae contained only homologous genes for *CDKA1*, which are associated with cell division and the cell cycle, but lacked true RDGs specific to root development. In the second stage, the transition from aquatic to terrestrial plants, the RDG network initially formed in bryophytes and ferns. The number of RDGs significantly increased, and genes including *PINs*, *PLTs*, *SHRs*, and *SCRs*, which are related to the establishment of root infrastructure, emerged. Among these, the newly added genes were primarily associated with primary and LR, while fewer genes related to RH appeared, resulting in a relatively simple RDG network. In the third stage, the RDG network was further refined. The number of RDGs increased further in both gymnosperms and angiosperms, and the networks for PRs and LRs became more extensive. Simultaneously, due to the increase in the number and diversity of RH-related genes, the RDG network for RHs was gradually enriched and refined. RDGs differ between dicotyledonous and monocotyledonous plants, and the genus *Brassica* within dicotyledonous plants encompasses all types of RDG networks.

Building on the premise that U’s triangle serves as a robust model for studying gene expansion, we further identified RDGs across the six target species. The results revealed that these RDGs are highly conserved and stable within their evolutionary relationships. Although the majority of RDGs remained evolutionarily stable, a subset of genes underwent tandem repeats, such as the copy number increase of *CDKA1s* and the copy number decrease of *TRYs* observed in *B. napus* (Table S3).

### 2.3. Linear Relationship Analysis of RDGs in B. napus

**Figure 3:**
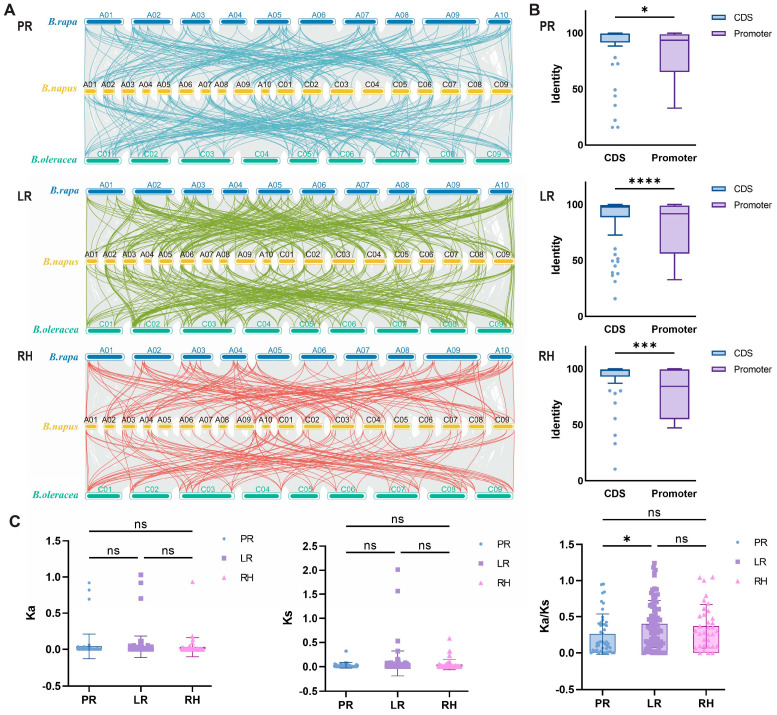
Evolutionary analysis of RDGs in *B. napus*. (**A**) Linear relationship analysis of *B. napus* RDGs and homologous genes in *B. rapa* and *B. oleracea* genomes. (**B**) Comparison of CDS and promoter identity in PR, LR, and RH. *p* value was calculated by *t* tests. *, ***, and **** mean significant differences at the 0.05, 0.001, and 0.0001 probability levels, respectively. (**C**) Comparison of Ka, Ks, and Ka/Ks values among PR, LR, and RH. *p* value was calculated by one-way ANOVA. * mean significant differences at the 0.05 probability level. ns means no significance.

To further explore the genetic relationships within the *B. napus* genome, which is composed of AACC subgenomes, we conducted an intraspecific collinearity analysis (Figure 3A). Preliminary analysis of the evolutionary mechanisms revealed that RDGs had a total of 486 linear pairs between the *B. napus* and *B. oleracea* genomes, and 446 linear pairs between the *B. napus* and *B. rapa* genomes. Among the RDGs in the PR network, approximately 34.16% (166 pairs) of linear relationships were found between *B. napus* and *B. rapa* genomes, and about 38.79% (173 pairs) between *B. napus* and *B. oleracea* genomes. Within the LR network, the distribution of root development genes (RDGs) revealed that approximately 53.29% (259 pairs) were identified between *B. napus* and *B. rapa* genomes, and 55.61% (248 pairs) were identified between *B. napus* and *B. oleracea* genomes. Among the RDGs in the RH network, the linear associations between *B. napus* and *B. rapa* genomes accounted for approximately 27.16% (132 pairs), and that between *B. napus* and *B. oleracea* genomes accounted for about 22.20% (99 pairs). These results indicate a significant degree of covariance between *B. napus* and both of its parental species. The ratio of genes from the two parental backgrounds in the LR network was nearly equal (1:1). In contrast, the PR network retained a higher proportion of genes from the *B. oleracea* and *B. rapa* background.

Meanwhile, we analyzed the sequence identity of 204 duplicate gene pairs generated via WGD (Figure 3B). Approximately 90.96% (185 genes) of the duplicated genes exhibited ≥60% sequence identity at the CDS level. However, sequence identity was significantly lower in the promoter regions, with only 74.02% (151 genes) of the duplicated genes showing ≥60% sequence identity. This suggests that the evolution of the RDG network in *B. napus* primarily involves functional differentiation and regulatory changes in promoter regions, while the coding regions remain relatively conserved and exhibit evolutionary lagging.

The Ka/Ks ratio was used to evaluate selection pressure during molecular evolution. Results indicated that most RDGs (~68.75%) underwent purifying selection in *B. napus*, while a smaller proportion (~31.25%) underwent neutral selection (Figure 3C). These findings indicate that the evolution of RDGs is primarily driven by purifying selection or neutral selection.

### 2.4. Potential Transcriptional Regulation Profile of RDGs in B. napus

**Figure 4:**
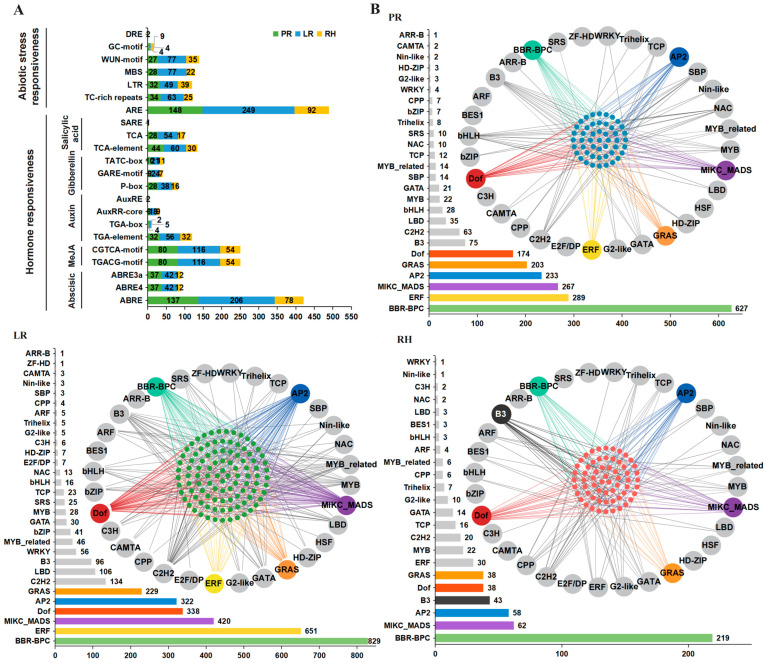
Transcription and post-transcription regulation analysis of RDGs. (**A**) The 22 types of important CREs in the promoter regions of RDGs. The X-axis represents the number of RDGs. (**B**) The potential regulatory interaction between TF and RDGs. Large circles represent predicted TF genes. Small blue circles represent genes in the PR network, small green circles represent genes in the LR network, and small red circles represent genes in the RH network. The green, yellow, purple, red, blue, orange, and black lines indicate the regulation of RIDGs by BBR-BPC, ERF, MIKC_MADS, Dof, AP2, GRAS, and B3 genes, respectively.

We identified 115 types of 29,401 *cis*-regulatory elements (CREs) in the promoter regions (−1500 bp) of RDGs in *B. napus* using the PlantCARE online software (https://bioinformatics.psb.ugent.be/webtools/plantcare/html/, accessed on 2 September 2022) (Figure 4A). In addition to the common basic core *cis*-elements (e.g., TA-TA-box, CAAT-box, and GC-box) and light-responsive *cis*-elements (e.g., G-box and GATA-motif), we identified a total of 937 *cis*-elements associated with seven abiotic stress-responsive *cis*-elements, including low-temperature-responsive *cis*-elements, salt-stress-responsive *cis*-elements, and injury-responsive *cis*-elements. Additionally, we identified 1514 *cis*-elements related to 15 hormone responses across five categories, including growth hormone response *cis*-elements, abscisic acid response *cis*-elements, gibberellic acid response *cis*-elements, salicylic acid response *cis*-elements, and methyl jasmonate response *cis*-elements.

Furthermore, we predicted the TF binding sites in the promoter regions of RDGs in *B. napus*. The results showed that there were 5065 pairs of potential transcriptional regulatory relationships between 333 genes from 31 TF families and RDGs in *B. napus* (Figure 4B). Among these, the RDG network might appear to be complexly regulated by a large number of upstream TF, primarily BBR-BPC, MIKC_MADS, AP2, and GRAS TF families. Interestingly, in the PR network, the GRAS family regulates 23 RDGs with only one member, while the 66 members of the ERF family regulate only 14 RDGs. This quantitative regulation relationship also exists in RH and LR networks, suggesting that different TF families have a more complex approach to regulating root systems.

### 2.5. Analysis of DEGs and LN Stress Expression Profiles of RDGs in B. napus

**Figure 5:**
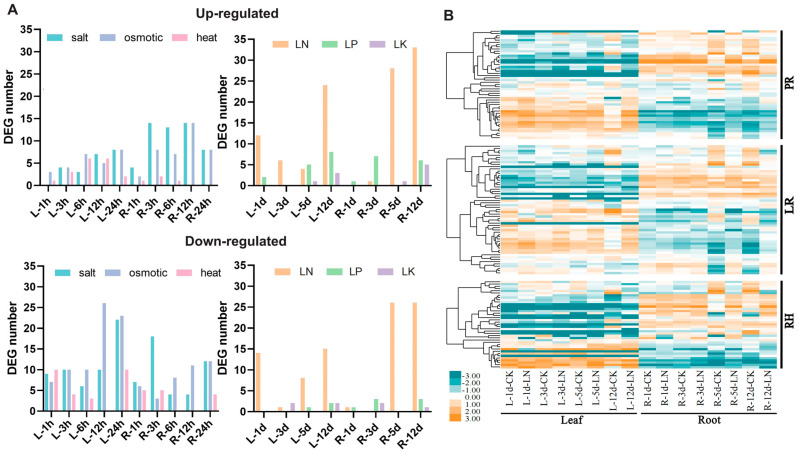
Expression of RDGs under abiotic and nutrient stress. (**A**) Number of DEGs in RDGs under abiotic and nutrient stresses. 1 h, 3 h, 6 h, 12 h, 24 h, 1 d, 3 d, 5 d, and 12 d represent the time elapsed after different treatments. L: leaf; R: root. (**B**) LN stress expression profile of RDGs in *B. napus*. There are 130 genes in RDGs with detectable expression levels in *B. napus* roots and/or leaves (FPKM ≥ 1). In the color bar, orange represents a high level of expression, and yellow represents little or no expression. LN: low nitrogen treatment.

We utilized RNA-seq datasets from the BnaGADB website (http://www.bnagadb.cn/, accessed on 1 September 2022) for ZS11 seedling leaves and roots under low nitrogen (LN), low phosphorus (LP), and low potassium (LK) treatments, and from the BnTIR website (http://yanglab.hzau.edu.cn/BnTIR/, accessed on 1 September 2022) to obtain data related to heat stress, salt stress, and osmotic stress. The number of differentially expressed genes (DEGs) compared to the control group under different treatment conditions was quantified (Figure 5A). The results showed that the number of up-regulated genes in roots was higher than leaves under nutrient stress conditions. Notably, the number of up-regulated genes in roots under LN increased over time.

Based on the DEGs analysis, we further investigated the expression patterns under LN (Figure 5B). In the LN stress expression profile, 109 (~50.00%) of the 218 RDGs in *B. napus* exhibited differential expression in roots (FC ≥ 2, FDR < 0.01). Among the PR network, 23 (~51.11%) genes were up-regulated, and 8 (~17.78%) genes were down-regulated. In the LR network, 10 (~21.28%) genes were up-regulated, and 27 (~57.45%) genes were down-regulated. In the RH network, 19 (~59.38%) genes were up-regulated, and 5 (~15.63%) genes were down-regulated. These results indicate that half of the RDGs in *B. napus* were differentially expressed under LN stress, with more DEGs up-regulated in PR and more DEGs down-regulated in LR. Thus, RDGs in *B. napus* widely respond to LN stress, with expression trends varying across the RDG network in different root networks.

### 2.6. Functional Validation of the BnaSHR-6 Gene in Arabidopsis Mutants

**Figure 6:**
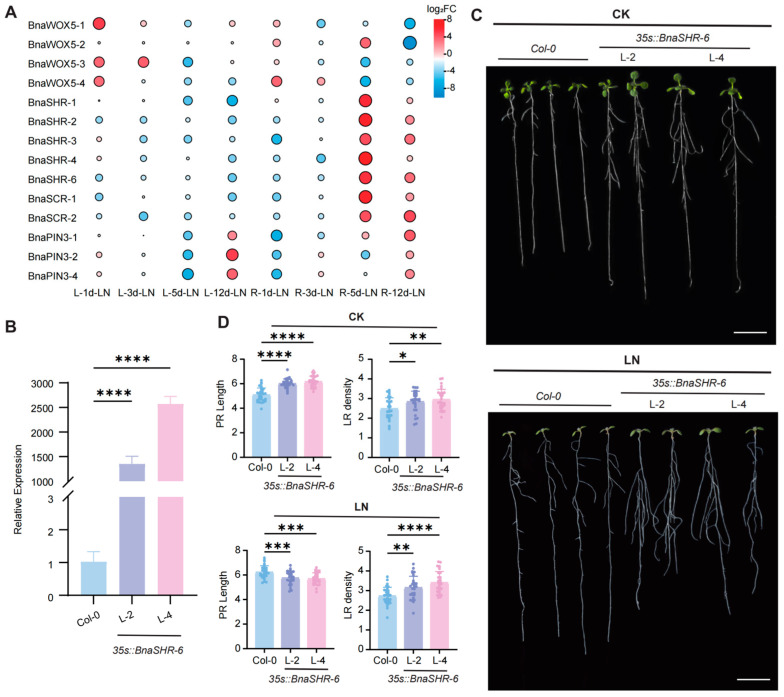
Phenotype analyses of the *BnaSHR-6* transgenic lines under CK and LN treatments in *Arabidopsis*. CK: full nutrition; LN: low nitrogen. (**A**) DEGs among shared genes in the PR and LR networks under LN stress. L: leaf; R: root. (**B**) The gene expression of *BnaSHR-6* in *35Sp:: BnaSHR-6* transgenic lines and *Col-0*. Data from 10-day-old seedlings. n = 3 biological replicates, *p* value was calculated by one-way ANOVA. **** mean significant differences at the 0.0001 probability level. L: line. (**C**) Phenotypic analysis of *Arabidopsis* under CK and LN. (**D**) Statistical data of the PR length and LR density at *Col-0* and *35Sp:: BnaSHR-6* transgenic lines. Data from 12-day-old seedlings. n ≥ 30, *p* value was calculated by one-way ANOVA. *, **, ***, and **** mean significant differences at the 0.05, 0.01, 0.001, and 0.0001 probability levels, respectively. Scale bar: 1 cm.

The analysis of 26 genes common to both PR and LR networks revealed differential expression of 14 genes under low nitrogen (LN) stress. Among these, 10 genes were up-regulated, and 4 were down-regulated (Figure 6A). Notably, of the 10 up-regulated genes, *BnaSHR-2*, *BnaSHR-4*, *BnaSHR-6*, *BnaSCR-1*, and *BnaSCR-2* demonstrated up-regulation on both days 5 and 12. The remaining genes were up-regulated exclusively on day 5 or day 12. Given that *BnaSHR-6* displayed relatively higher expression levels in *B. napus* roots and exhibited higher and more stable up-regulation, it was considered a candidate gene for further molecular function validation studies.

To explore the function of *BnaSHR-6*, we generated *35Sp::BnaSHR-6* transgenic lines and cultured them on both standard Hoagland’s solid medium and LN media, alongside the wild-type (*Col-0*). Under control conditions, the *35Sp::BnaSHR-6* line showed longer PR length and greater LR densities compared to *Col-0* (Figure 6C). This suggests that *BnaSHR-6* positively regulates both PR and LR development. Phenotypic analyses under LN conditions revealed increased PR and LR lengths in *Col-0*, whereas the *35Sp::BnaSHR-6* line exhibited no significant change in PR length but showed an increase in LR length. This indicates that *BnaSHR-6* responds positively and exhibits resistance to LN stress. These findings suggest that *BnaSHR-6* is up-regulated under LN conditions, supporting its role in root development under such conditions.

## 3. Discussion

### 3.1. Genome Evolution and Functional Diversification of RDGs in B. napus

*B. napus* is an allotetraploid formed by the hybridization between *B. rapa* and *B. oleracea*, both of which have experienced ancestral whole-genome triplication (WGT) events common in Brassicaceae [29,30]. Given the high conservation of homologous genomic regions, the gene number in *B. napus* is theoretically approximately six times that of *Arabidopsis*. For example, the 53 RDGs identified in *Arabidopsis* should expand to approximately 318 genes in *B. napus* through polyploidization. However, only 218 orthologs were identified, indicating that approximately one-third of genes were lost through large-scale homologous recombination following WGT. The diploid progenitors *B. rapa* and *B. oleracea*, contain 103 and 107 root initiation-related genes, respectively. In the *B. napus* subgenomes, 106 genes are located in the A_n_ subgenome and 110 in the C_n_ subgenome, with two remaining unknown, showing varying retention rates between subgenomes, likely due to subgenome dominance selection. Sequence analysis of the 218 genes in *B. napus* revealed that their CDS are highly conserved (>90% identity), while promoter sequences exhibit significant diversity. This suggests that the functional diversification of these genes may enhance environmental adaptability during root development. Additionally, frequent small-scale duplication events (17.43% tandem repeats) and positive selection in promoter regions were found to continuously introduce new variation into the RDG network.

### 3.2. Expression Patterns of RDGs in B. napus

Analysis of DEGs related to RDGs in *B. napus* under abiotic and nutrient stresses revealed that DEGs were present under almost all stress conditions, suggesting that these genes may be involved in regulating root stress adaptation and nutrient acquisition. For example, studies have shown that the transcription factors ARF7 and ARF19 respond to phosphorus deficiency by positively regulating *PHR1*, a key regulator of phosphorus starvation responses in *Arabidopsis* roots [31]. In *Arabidopsis*, *GL2* directly modulates *ETO1* expression and function under LN, LP, or LK stress [23,32]. *TCP20* binds to the DNA of over 100 nitrate-regulated genes, playing a crucial role in systemic nitrate responses [33,34]. Notably, a relatively higher number of DEGs was observed under LN stress, suggesting that RDGs under LN stress may positively influence root growth and development, indicating potential applications in agriculture. Among the six genes consistently up-regulated in roots at both 5 and 12 days of LN treatment, all belonged to the GRAS family. Two family members, *SHR* and *SCR*, interact to specify endodermal cell identity and regulate root apical meristem establishment and maintenance. *BnaSHR-6* exhibited higher expression levels and more stable up-regulation under LN, implying its potential role in modulating root growth under LN conditions.

### 3.3. The Critical Function of BnaSHR-6 in B. napus

Short Root (SHR) acts as a mobile TF and plays a crucial role in regulating the asymmetric division of root cortex/endodermis stem cells, maintaining the activity of the root apical meristematic [30,31]. The duplication of the *SHR* gene is widespread, with extensive homologs found in *Oryza sativa*, *Brachypodium distachyon*, *Zea maize*, *Medicago truncatula*, and various other plant species [35]. In *B. napus*, *BnaSHR-6* was identified as a crucial factor responsive to LN conditions. Under control conditions, the *35Sp::BnaSHR-6* transgenic line showed increased PR length and higher LR density compared to the wild type (*Col-0)*. Under LN conditions, *Col-0* exhibited a significant increase in both PR and LR length, consistent with previous findings [32]. In contrast, the *35Sp::BnaSHR-6* line did not show a notable change in PR length but did show a significant increase in LR density. This further confirms that *BnaSHR-6* positively responds to LN, influencing the development of both PR and LR. The up-regulation of *BnaSHR-6* under LN highlights its essential role in maintaining root architecture under LN stress. As a key regulator of root development under LN, *BnaSHR-6* may also preliminarily affect the growth of aboveground leaves. Under control conditions, the aboveground leaves of the *35Sp::BnaSHR-6* lines showed enhanced growth compared to *Col-0*. Under LN stress, transgenic lines maintained relatively superior leaf growth despite overall reduction. Given that SHR is expressed in vascular tissues of Arabidopsis (inflorescences, stems, leaves) and regulates leaf cell proliferation and vascularization [36,37], we hypothesize that *BnaSHR-6* could extend its regulatory role beyond roots to modulate leaf development under stress conditions. This potential mechanism warrants further validation in *B. napus*.

## 4. Materials and Methods

### 4.1. Identification and Subcellular Localization of RDGs in B. napus

To identify RDGs in *B. napus*, we conducted a detailed review of the relevant literature reports and summarized the RDGs that have been functionally defined in *Arabidopsis*, and retrieved the protein and CDS sequences of these genes from the TAIR website (https://www.arabidopsis.org/, accessed on 1 September 2022) and the Phytozome v12.1 database (http://www.Phytozome.net/, accessed on 1 September 2022) [38]. The genome database of the rapeseed variety “ZS11” was searched for homology using BLASTP. The Expectation Value threshold was set to 0.0001, and the rest of the parameters were set by default [39,40]. Then, multiple sequence alignment was performed using MAFFT version 7 (https://mafft.cbrc.jp/alignment/server/, accessed on 1 September 2022), and after deletion of sequences with large gaps (>60%) and redundancies, all homologous genes in the RDG network in *B. napus* were analyzed and obtained in combination with sequence similarity (>80%) [41]. The accuracy of the data was further checked by predicting and analyzing the protein structures encoded by the candidate genes using SMART (https://smart.embl.de/, accessed on 1 September 2022) and ExPaSy websites (https://www.expasy.org/, accessed on 1 September 2022). RDGs in *B. rapa* and *B. oleracea* were also characterized using the methods described above. The predicted subcellular localization of root initial development-related proteins in *B. napus* was analyzed using the Plant-mPLoc (http://www.csbio.sjtu.edu.cn/bioinf/plant-multi/, accessed on 1 September 2022) and WoLF PSORT (https://wolfpsort.hgc.jp, accessed on 1 September 2022) websites [42,43].

### 4.2. Chromosomal Localization and Collinearity Analysis of RDGs in B. napus

This study employed the GBROWSE tool from the BnPIR website (http://cbi.hzau.edu.cn/cgi-bin/bnapus/gb2/gbrowse/ZS11v0/, accessed on 1 September 2022) to conduct a comprehensive analysis of the chromosomal localization of RDGs in *B. napus*, and the chromosome distributions of RDGs were drawn using MapChart 2.3.2 software [44,45]. The One Step MCScanX plugin within the TBtool v2.056 software was used to perform an in-depth analysis and visualization of the collinearity between RDGs in ZS11 and those in the genomes of *B. rapa*, *B. oleracea*, and *B. napus* [46]. The duplication events of RDGs were defined based on the collinearity relationship.

### 4.3. Analysis of Amplification and Evolutionary Mechanism of RDGs in B. napus

The CDS sequences and promoter sequences (−1500 bp) of RDGs in *B. napus* were obtained using the BnPIR website (http://cbi.hzau.edu.cn/cgi-bin/bnapus//blast, accessed on 1 September 2022) [44]. Similarity and identity between duplicated gene pairs across CDS and promoter sequences were subsequently analyzed using MatGAT 2.01 software, thus assessing the conservation of gene duplications [47]. Also, based on the obtained CDS sequences of RDGs and the replication relationships among RDGs in *B. napus*, selection pressure analysis was performed using KaKs_Calculator 2.0 software (https://bigd.big.ac.cn/tools/kaks, accessed on 1 September 2022) to calculate the model with LWL [48].

### 4.4. Identification of RDGs in the Plant Kingdom

Based on the protein sequences of the RDGs in *Arabidopsis*, homology searches of the remaining representative species of the Kingdom Plantae and representative species of Brassicaceae were performed using BLASTP to identify the RDGs in the corresponding species. Among them, the genomic data for *Chlamydomonas reinhardtii*, *Volvox carteri*, *Physcomitrella patens*, *Selaginella moellendorffii*, *Amborella trichopoda*, *Oryza sativa*, *Zea mays*, *Aquilegia coerulea*, *Solanum tuberosum*, *M. truncatula*, and *Populus trichocarpa* were obtained from the Phytozome v12.1 database (https://phytozome-next.jgi.doe.gov, accessed on 1 September 2022). *Picea abies* genomic data was obtained from the TreeGenes website (https://treegenesdb.org/FTP, accessed on 1 September 2022). *Brassica nigra*, *Brassica juncea*, *Brassica carinata*, *B. napus* were obtained from the BnTIR website (http://yanglab.hzau.edu.cn/BnTIR, accessed on 1 September 2022). Data processing and preliminary analysis of RDGs in the above species were performed using Microsoft Excel software.

### 4.5. Functional Prediction Analysis of RDGs in B. napus

To elucidate the regulatory mechanism of RDGs in *B. napus*, we used the PlantTFDB website (http://planttfdb.gao-lab.org, accessed on 1 September 2022) to predict the potential transcription factor binding sites (*p*-value ≤ 1 ×10^−6^) of the promoter sequences (−1500 bp) of RDGs in *B. napus* [49]. *Cis*-elements of the promoter sequences (−1500 bp) of RDGs in *B. napus* were predicted using the PlantCARE website (http://bioinformatics.psb.ugent.be/webtools/plantcare/html, accessed on 1 September 2022) [50]. Cytoscape 3.8.2 software was used to visualize the prediction results [51].

### 4.6. Expression Pattern Analysis of RDGs in B. napus

To explore the nutrient-responsive expression patterns of RDGs in *B. napus*, the RNA-seq dataset of ZS11 seedling leaves and roots under LN, LP, and LK treatments was obtained from the BnaGADB website (http://www.bnagadb.cn/, accessed on 1 September 2022). The expression patterns were further analyzed utilizing abiotic stress expression profile data sourced from the BnTIR website (http://yanglab.hzau.edu.cn/BnTIR/, accessed on 1 September 2022), which encompasses profiles related to heat stress, salt stress, and osmotic stress. We focused on RDGs exhibiting maximum fragments per kilobase of transcript per million mapped reads (FPKM) values greater than or equal to 1 across all samples for subsequent differential expression analysis. We employed the DESeq2 package to conduct this analysis, applying a threshold of log2 (fold change) ≥ 1 and *p*-value < 0.05 to identify significantly differentially expressed genes. Data processing and subsequent analysis were performed using Microsoft Excel 2022. The expression data of the above groups were processed separately using Cluster 3.0 and visualized using Java Treeview 3.0 and TBtools v2.121 [52,53].

### 4.7. Phenotypic Analysis of Transgenic Arabidopsis

We used the *pEASY-BnaSHR-6* vector as a template and designed specific primers (F: 5′-ATGGATACTCTCTTTAGACTAGTCAGTCTCC-3′; R: 5′-CGTTGGCCGCCACGCACTG-3′) to amplify the *BnaSHR-6* gene fragment by PCR. The amplified fragment was digested with *Kpn* I (N-terminal) and *BamH* I (C-terminal) restriction enzymes, and the full-length CDS of *BnaSHR-6* was cloned into the middle of the *CaMV35Sp* promoter and the *Flag* gene by T4-DNA ligase in the pC1300-Flag vector to construct the *35Sp:: BnaSHR-6-Flag* vector. Then, we conducted a phenotypic analysis on the *Arabidopsis* line obtained, including the wild type (*Col-0*) and transgenic *Arabidopsis* (*35Sp::BnaSHR-6*). Seeds were surface sterilized with 75% ethanol for 15 min, then washed 3–5 times with sterile deionized water (ddH_2_O) and sown on full nutrient Hoagland medium and LN solid media (Table S4). After 3 days of 4 °C vernalization, they were planted in an artificial climate culture chamber. The specific conditions were set at an illumination intensity of 20,000 Lux, a 16 h photoperiod at 23 °C, followed by an 8 h dark period at 20 °C, and 70% humidity. Transgenic *Arabidopsis* and *Col-0* were germinated in Hoagland medium for four days in the same environment and then transferred to LN medium for eight days for phenotypic analysis. PR and LR were observed and measured for Transgenic *Arabidopsis* and *Col-0*, and the resulting data were statistically analyzed and visualized using GraphPad Prism version 9.5 software.

### 4.8. RT-qPCR Analysis of 35Sp::BnaSHR-6 Overexpressing Transgenic Arabidopsis

The qRT-PCR method was applied to analyze the expression levels of *35Sp::BnSHR-6* and *Col-0*, using *AtActin* as control. Seedlings grown on full nutrient Hoagland medium for 10 days were used. Each treatment was performed with three biological replicates, each replicate containing five plants. The primers of *35Sp::BnSHR-6* and AtActin used in this analysis are listed in Table S5. The RNA simple total RNA Extraction Kit (TIANGEN, Beijing, China) was used to extract the total RNA in each sample. Then, the quality and concentration of the total RNA were examined using 1% gel electrophoresis and a Thermo Fisher ScientificTM spectrophotometer (Thermo, Beijing, China), respectively. Subsequently, about 1.0 μg of the total RNA was applied to synthesize first-strand cDNA in a 20 μL reaction system, according to the manufacturer’s instructions for HiScript^®^ III RT SuperMix for qPCR (+gDNA wiper) HiScript^®^ III RT kit (Vazyme, Nanjing, China). The cDNA concentration was diluted to 35 ng/μL for subsequent experiments. The Taq Pro Universal SYBR qPCR Master Mix Kit (Vazyme, Nanjing, China) was used to perform real-time PCR analysis in a CFX Connect™ Real-Time System (Bio-Rad, Hercules, CA, USA). The cDNA was amplified using a 40-cycle program (95 °C, 5 s; 58 °C, 20 s per cycle) following denaturation. Expression levels were calculated as the mean signal intensity across the three biological replicates using the 2^−ΔΔCt^ method. One-way ANOVA analyses of variance were used to assess the difference in the expression level of each gene.

## 5. Conclusions

In this study, a total of 218 RDGs were identified within the *B. napus* genome, and the RDG network in *B. napus* was systematically mapped and constructed. We conducted a comprehensive analysis of the amplification and evolutionary mechanisms, phyletic distribution patterns, transcriptional regulation network characteristics, and expression properties of this network. Notably, we identified the gene *BnaSHR-6*, which is part of both the PR and LR networks and exhibits significant up-regulation under LN stress. In this study, a homology search in *B. napus* based on known RDGs in *Arabidopsis* was performed with high conservation and reference value. However, the exclusive reliance on *Arabidopsis* homologs may have led to the omission of RDGs specific to *B. napus*. To mitigate this limitation, future research should incorporate de novo transcriptomic analyses of root development stages alongside proteomic studies. The integration of multi-omics datasets with forward genetics has the potential to delineate a detailed roadmap of root development for specific species. Ultimately, this systems biology framework can expedite the application of RDGs from fundamental research to the precision breeding of high-yielding rapeseed cultivars, thereby providing both theoretical insights and practical genetic resources essential for sustainable agriculture.

## Data Availability

The original contributions presented in this study are included in the article/Supplementary Material. Further inquiries can be directed to the corresponding authors.

## Supplementary Materials

The following supporting information can be downloaded at: https://www.mdpi.com/article/10.3390/plants14121842/s1, Table S1: Features of the Root development genes (RDGs) from *Brassica napus* and *Arabidopsis thaliana* identified in this study. Table S2: Specific distribution of RDGs in 12 typical plants. Table S3: Specific distribution of RDGs in six species in the U’s triangle. Table S4: List of the normal and LN Hoagland’s components used in this study. Figure S1: Subcellular localization of root development-related proteins in *B. napus*. Figure S2: Number of RDGs on each chromosome.
