# Supplementary material for: The Root Development Genes (RDGs) Network in Brassica napus and the Role of BnaSHR-6 in Response to Low Nitrogen
Source: Plants (Basel). 2025 Jun 15;14(12):1842. doi: 10.3390/plants14121842 (PMC12196633; doi:10.3390/plants14121842)
Supplement: Supplementary file 1 [file plants-14-01842-s001.zip › plants-3680067-supplementary/RDGs-supplementary materials/Figure S1.pdf]

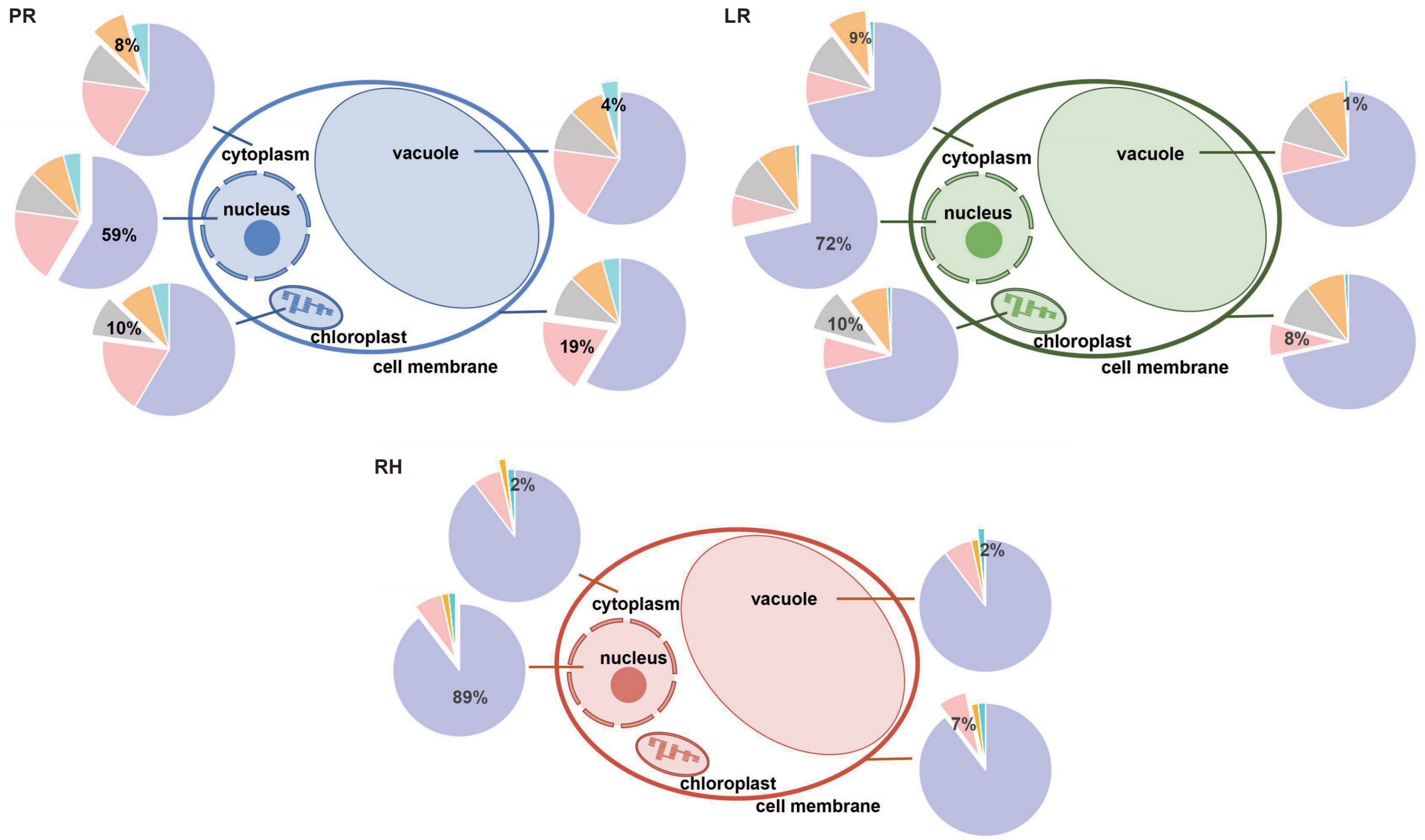

FigureS1. Predicted distribution of subcellular localization of proteins related to root development in *B. napus*.The PR network is represented as blue, the LR network is represented as green, the RH network is represented as red.
