# Supplementary material for: The Root Development Genes (RDGs) Network in Brassica napus and the Role of BnaSHR-6 in Response to Low Nitrogen
Source: Plants (Basel). 2025 Jun 15;14(12):1842. doi: 10.3390/plants14121842 (PMC12196633; doi:10.3390/plants14121842)
Supplement: Supplementary file 1 [file plants-14-01842-s001.zip › plants-3680067-supplementary/RDGs-supplementary materials/Figure S2.pdf]

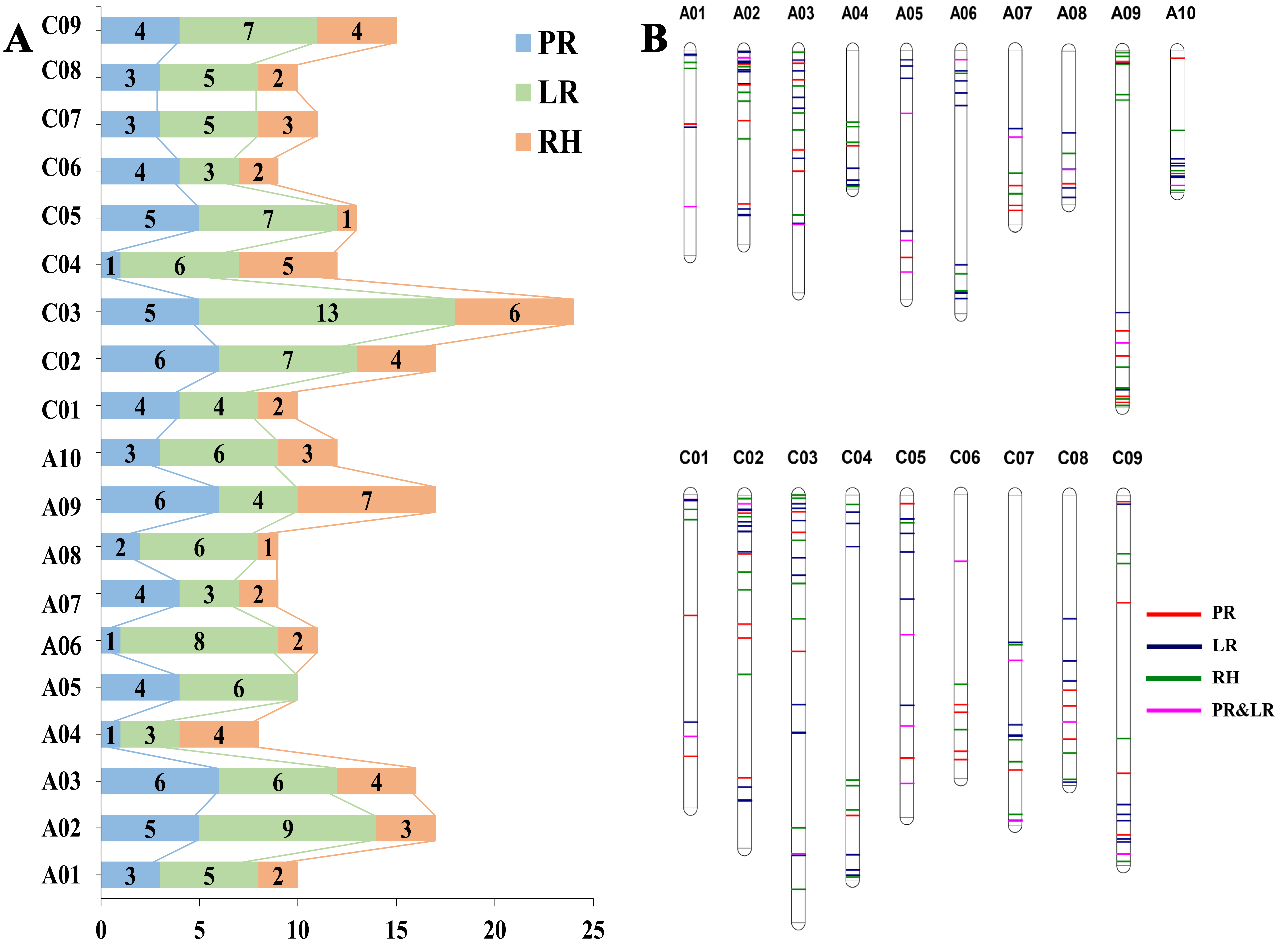

FigureS2.Chromosome analysis of RDGs in B. *napus*(A)Chromosome mapping of RDGs in *B. napus.*(B)Number of K+ utilization genes on each chromosome.
